# Some Affections of the Gums

**Published:** 1888-10

**Authors:** Frank Lankester

**Affiliations:** England.


AMERICAN JOURNAL

—OF—

DENTAL SCIENCE.

VOL. XXII. THIRD SERIES—OCTOBER, 1888. NO. 6.

ARTICLE I.

### SOME AFFECTIONS OF THE GUMS.

BY FRANK LANKESTER, L. R. C. P., M. R. C. S., L. D. S. ENGLAND.

We will now pass on to consider some of the *constitutional affections*, and will take, first, that somewhat rare affection known as *Purpura hæmorrhagica*, in which disease the gums are more or less affected. It requires medical treatment, so I will not do more than mention it here. It occurs chiefly in the young and debilitated, and is characterised by extravasations of blood in the form of red points or *patechiæ* beneath the skin and mucous membranes, including the tongue, lips and gums. These resemble somewhat the small extravasations of blood that one not infrequently sees after having had the skin pinched unpleasantly hard, as with a pair of pliers, &c. There is always a tendency to hæmorrhage from all the mucous surfaces of the body, and it is only its rare occurrence that makes the disease comparatively unimportant. It may prove serious from the persistance of the hæmorrhage and the difficulty of arresting the same.

Another disease now happily also rare is *True Scurvy*, in which also we find extravasations beneath the gums. At first, merely the edges of the gums become somewhat swollen; the condition increases and lobulated masses form and rise up around and between the teeth, so that the latter may even be hidden. The gums get very spongy, insensitive, and of a blotchy deep red or livid colour, and they are very apt to bleed; indeed, in severe cases blood oozes continually from the gums. Ulceration and sloughing may supervene, and lay bare the necks of the teeth. The latter may get loose and fall out. The breath is generally very fœtid and there is a liability to submucous hæmorrhage. It occurs chiefly in navvies who live badly and take but little vegetable food. The *treatment* belongs to the general practitioner, and consists first in remedying the cause, whilst locally antiseptic and astringent mouthwashes will help the swollen state of the gums to quickly subside.

There is another condition of the gums known as *False scurvy*, which is frequently met with in persons who neglect to clean their teeth, and are in feeble health. Salter describes it as consisting essentially of a vascular dilation of the papillary and other capillaries, with a general thickening of the gum itself. It is a condition that can soon be remedied by improving the health and the use of a tooth-brush with astringent mouth-washes to get the mouth and teeth clean and sweet. The gums will then soon resume their normal appearance.

*Addison's Disease* we will briefly consider. It is a comparatively rare disease and is characterised by various nervous and dyspeptic symptoms, together with an ever-increasing debility and a most renarkable pigmentation or bronzing of the skin and mucous membranes. The skin, especially on exposed surfaces, assumes a deep brown mulatto tint whilst on the tongue, gums and buccal mucous membrane there are found irregular patches of pigmentations of a dark brown or blackish colour, and which are quite characteristic of the disease, and could not well escape the notice of anyone examining the mouth. It occurs chiefly in males between ten and fifty years of age, and it always ends fatally, and commonly within a year. The treatment is purely palliative. I merely mention the disease here to-night as a rare abnormal condition of the gums, as a curiosity, so to speak.

I have mentioned four metallic poisons that exert an influence on the gums. We will take *Mercurial Poisoning* first. Mercury enters the system very readily, both by the stomach, skin and the lungs. It is taken very largely as a medicine, and is much used in various Arts and manufactures, such as glass, &c., &c. Hence we not infrequently meet with cases of mercurialism, *i. e.*, mercurial poisoning. One of the earliest indications of this is its effect on the gums, which generally give the first token in the form of a red line running along their margins; this is soon followed by much tenderness, swelling and pulpy thickening of a dark red colour first appearing around the incisor teeth. The gums finally retract from the teeth, the tongue becomes swollen and furred and the breath fœtid. There is, too, a metallic taste present in the mouth. Besides this there is more or less profuse salivation, and the glands themselves become swollen and painful. If the inflammation goes on ulceration may supervene and large portions of the gums and cheeks may thus be destroyed. The teeth will become loose and fall out, and the maxillæ even carious. The *treatment* belongs to the surgeon or physician. The administration of the mercury should be discontinued for a time. Locally astringent mouthwashes are useful, whilst internally tonics should be given. Where you wish to get the mercury quickly out of the system, iodide of potassium will be found the most useful drug, though it often produces increased salivation at first. We should not forget that when the patient is kept too long under its influence, very serious nervous and other affections may result, especially the so-called metallic *tremor* that affects all the muscles of the body. In some persons the effect of the drug is produced much more quickly than in others, and it has an especially debilitating effect on persons already exhausted by disease. Children under two years of age are rarely if ever salivated, but we must remember a peculiar malformation of the permanent teeth which M. Hutchinson has pointed out as being due to the incautious use of grey and other teething powders. The defect of development is said to be due to the stomatitis that is caused by the mercury, leading thereby, secondarily, to inflammation of the developing enamel organs. If for no other reason than this we ought to be familiar with the ill effects of mercurialism. As a query by the way, Ringer remarks that there are those who cannot take even the smallest doses of mercury without it causing toothache. Is this independent of caries ? Perhaps some gentleman may be able to confirm this statement, which I thought of sufficient interest to permit of its being mentioned here.

We must now pass on to the consideration of *Chronic Lead Poisoning* so far as it affects the gums. It is a condition that one might meet with occasionally and perhaps quite accidentally in private practice and it is well that we should be familiar with its appearance. In *plumbism* we get the "blue line on the gums." This was first described by Dr. Burton, and is due to the action of sulphuretted hydrogen on the lead ; the latter having been previously deposited in the substance of the gums, "black" sulphide of lead is formed, hence the discoloration. We have the development, then, of a narrow bluish or violet line along the free margins of the gums wherever the latter came into contact with the teeth. The sulphuretted hydrogen is formed by the decomposing matter accumulating around the necks of the teeth. Where there are no teeth there will be no place for the tartar or food to collect, and hence there will be no sulphuretted hydrogen, and therefore, also, no "blue line" formed. It appears first and is always most marked around the incisor teeth, especially the interdental portions, and this is particularly the case when the teeth are not kept clean. It may extend to the whole of the gums and even to the cheeks. The gums themselves are frequently retracted thereby, causing the teeth to appear elongated; the latter are sometimes discoloured, but whether this is in any way due to the lead or not I am uncertain. When present, as it almost always is, it forms an infallible diagnostic sign of the presence of lead in the system; it is, however, occasionally absent, or at least very faintly marked, when the teeth are kept scrupulously clean. In rare cases it has been developed in the course of a few hours by two or three medicinal doses of lead. It is, of course, mostly found in painters, plumbers, &c., and those who are being continually brought into contact with it; and such patients may yet be free from all other symptoms of plumbism. Where there are no teeth it may be quite absent. When we remember the many very serious digestive and nervous paralytic troubles that arise from chronic lead poisoning and the very many different ways in which lead may be quite ignorantly and accidentally taken into the system, it is, I think most advisable and indeed highly important, so far as the patient is concerned, that we should very carefully inquire into all cases in which, from the condition of the gums, there is the slightest suspicion that the patient is the subject of plumbism. If our inquiries as to his general health, habits, &c., only tend to confirm our suspicion, we should then most certainly warn our patient as to his possible condition and urge him to obtain medical advice on the point. I only lately came across such a case at this hospital. I regret to say I cannot show you the patient to-night. The blue line along the gums was very well marked and the teeth were in a dirty condition generally. On inquiry he told me he was a gasfitter, &c.

There are two other metals which affect the gums in a somewhat similar manner, but their rarity makes them unimportant, and I will not detain you long in describing them. The first I allude to is silver, which metal may be introduced into the system of those working is silver mines, or those who have been taking the nitrate of silver medicinally for some length of time. It leads gradually to a permanent discolouration of the skin, which becomes of a deep uniform leaden hue, and we frequently find that this is preceded by a dark brown line on the gums. The latter; therefore is of some value to the physician, as it indicates the necessity of stopping the administration of the drug before the *skin* becomes permanently stained. This could hardly be confounded with lead, for some definite history will be obtainable of the introduction of silver into the system in some form or other. The other metal is *Copper.* In copper poisoning a greenish line appears along the gums.

In persons of a *Scrofulous* habit, we may often notice a red line running along the margins of the gums. This, too, may be frequently seen in debilitated and other conditions. Salter relates a most interesting case of a very chronic and extensive ulceration of the gum and palate. The surface was irregular and granulating, the ulceration being quite superficial. The teeth were loose, and there was no tendency for the affection to heal. The patient's appearance and history at once gave the clue to the cause of this particular diseased condition. His family had shown marked indications of scrofula, and he himself was the very embodiment of it. He was sixteen years of age, and at time of applying for advice the patient was suffering from a scrofulous ulcer on the arm, whilst the arms and legs were covered with scars the result of previous ulcers. It was a most typical example of a constitutional affection, manifesting itself locally in the form of an ulceration within the mouth. A short time ago, one of our members sent a case up to this hospital to obtain the surgeon's opinion and advice in the matter. I saw the case with Mr. W. Weiss. The patient was about twenty years of age, and gave a very strong family history of phthisis. She was evidently of a strumous habit herself, there being scars on the neck and obstruction of the nasal ducts, &c., besides a general debilitated condition. About ten months previously, patient said she first noticed a few white and red spots on the gum, situated over the upper left lateral root. The condition of gum has been very gradually spreading since then. The gums were very apt to bleed. On examining the gums, there was a small area over the upper incisor roots, which presented a very spongy and swollen condition, was of loose texture, and was covered with small papillæ. Tenderness and increased vascularity were well marked, the slightest touch leading to rather free oozing of blood. Along their margins was a narrow red line, whilst towards the sulcus they were of a pale colour. The teeth were beautifully white and healthy, and quite clean. The palate, also, was normal. There was a continuous slight discharge, and on pressure pus oozed up near the sulcus. Here a fine probe could be passed down on to a small spot of bare bone. We came to the conclusion that it was probably a case of caries of the alveolus occuring in a strumous subject, and that the condition of the gum was secondary to it and caused by the irritation of the constant discharge, &c., from the diseased bone beneath it. I recommended her to syringe it frequently with boracic acid lotion, and to take Easton's syrup internally, and endeavour to improve her general health as much as possible. She promised to let me know how she progressed, but I have not yet heard from her.

I much regret that time fails me to say anything about the various forms of stomatitis that are of such frequent occurrence and that arise from so many different causes. The syphilitic are specially worthy of our most careful consideration and attention. Quite apart from their frequency they are of very considerable importance, and this is especially the case when present in the mouths of children during the period of developing of the permanent teeth. I will only just mention a case of *Ulcerative Stomatitis* that

I had under treatment about ten days ago, and from which I afterwards took this rough model which I mow pass round. It shows very well the great destruction of gum that has occured all along the margins, so that the necks of the teeth are laid bare for a considerable distance. Patient was a boy of about five years of age. Some time ago he had an attack of measles, and during the last eight or ten days the mouth has been very sore and inflamed, the gums bleeding on the slightest touch, whilst the breath has been very fœtid. On examining the mouth there was a red line running all along the gum margins; the necks of the teeth were bare and bathed in pus. There was a great destruction of tissue, and the gums generally were much swollen and inflamed, bleeding freely on touching them. I ordered the child to take six grains of chlorate potash internally three times a day, and to rinse the mouth out frequently with a gargle of the same salt, to which a little Condy was to be added. I saw him again four days later, when all inflammation had entirely disappeared, the mouth was nice and clean and the breath sweet; in fact, the patient was well and the mouth normal, excepting the destruction of tissue that had taken place.

I must now very humbly apologise for having detained you so long, but the subject which we have been considering covers a very much wider area than I at first thought it did; as it is I have not said a word about some of the most important affections. My chief object has been to bring under your notice those affections especially which are of some diagnostic value and importance, and I should have done better had I confined myself more strictly to this object. But I have simply not had time to get a real grasp of the subject, much less to arrange and put it in a concise and interesting form. I have, however, done my best in this matter during the short interval since we last met, and to night I am sure you have done your best to give me an attentive and most patient hearing, for which I am extremely grateful to you all.—*London Dental Record.*

